# Save Your Tears for the Toxicity Assays—Carbon Nanotubes Still Fooling Scientists

**DOI:** 10.1021/acsomega.4c08211

**Published:** 2025-02-03

**Authors:** Johanna Suni, Salli Valkama, Emilia Peltola

**Affiliations:** Department of Mechanical and Materials Engineering, University of Turku, Turku 20500, Finland

## Abstract

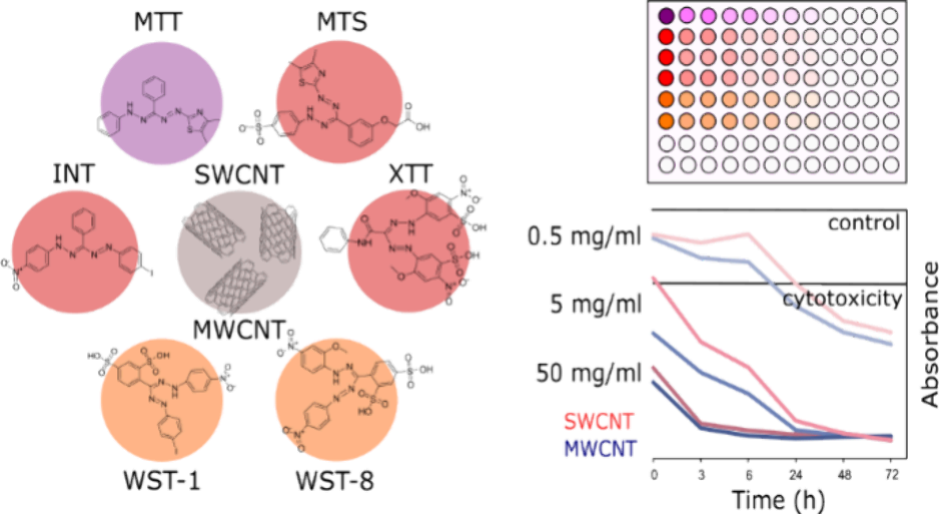

The extensive study of carbon nanotube (CNT) toxicity stems from their widespread application across various fields. The toxicity of CNTs is commonly assessed using cell viability assays based on tetrazolium salts, such as the MTT assay. ISO 10993–5 outlines the MTT assay and related *in vitro* cytotoxicity tests as international standards. However, nearly two decades ago, it was observed that MTT interacts with CNTs, potentially yielding inaccurate results. Despite this, the MTT assay remains the most widely used method for studying CNT toxicity *in vitro* today. Here, we demonstrate that six commonly used tetrazolium salts in cell viability assays—MTT, MTS, INT, XTT, WST-1, and WST-8— interfere with both single-walled nanotubes (SWCNTs) and multiwalled carbon nanotubes (MWCNTs). According to ISO 10993–5, cell viability percentages below 70% indicate cytotoxicity. At the standard testing duration of 3 h, the absorbance values in the presence of 5 mg/mL of either SWCNT or MWCNT decreased to below 70% relative to the control. At a lower concentration of 0.5 mg/mL, the effect was less pronounced, with the absorbance decreasing to an average of 84% compared to the control. Our results suggest that none of these cell viability assays alone offers a fully reliable method for evaluating CNT toxicity, especially with high CNT concentrations. Therefore, it is essential to carefully assess which *in vitro* methods are truly suitable for CNT toxicity studies.

## 1 Introduction

Nanotechnology is a field that has rapidly grown over the last few decades. One of the main areas within nanomaterials research is carbon-based nanomaterials, a family of carbon allotropes.^1^ Carbon nanotubes (CNTs) are cylindrical molecules composed of carbon atoms discovered in 1991 by Sumio IIjama.^2^ CNTs are categorized as single-walled carbon nanotubes (SWCNTs) and multiwalled carbon nanotubes (MWCNTs) based on the number of shells. Due to their unique mechanical, electrical, thermal, optical, and chemical properties, CNTs are promising materials for a wide range of applications in technology and medicine.^3^

Heister et al. and Murjani et al. reviewed the use of CNTs in various biological applications, including drug delivery, cell destruction agents, biosensors, prosthetic implants, and tissue scaffolds.^4,5^ Due to the large-scale production and use of CNTs in industry and commercial applications, their biocompatibility and toxicity have been extensively studied.^6−9^ Despite this, the biological interactions of CNTs remain unclear, and there are health risks associated with nanomaterials, as small particles can interact with biomolecules.^10,11^ In the body, CNTs have toxic effects, particularly in pulmonary tissue.^12−16^ Several factors have been found to affect CNT toxicity, including impurities, chemical and structural characteristics, and external factors.^7^ However, inconsistencies have emerged in *in vitro* studies, and challenges remain regarding the research methods used.

The most common way to study cell-material interactions *in vitro* is through spectroscopic analyses based on absorbance or fluorescence. Well-established assays for studying CNT toxicity include colorimetric cell viability assays based on tetrazolium salts, such as MTT, MTS, WST-1, and LDH assays.^17^ The international standard ISO 10993–5 also describes the MTT assay and related tests for *in vitro* cytotoxicity testing. MTT, MTS, and WST-1 assays measure cellular activity as an indicator of cell viability. In metabolically active cells, tetrazolium salts reduce to formazan crystal forms by NAD(P)H-dependent oxidoreductase enzymes. The LDH measures the activity of the cytoplasmic enzyme LDH (lactate dehydrogenase) released by damaged cells., which is quantified by measuring the reduction of the tetrazolium salt INT to formazan. To evaluate the toxicity of CNTs, the reduction in the associated absorption or fluorescent emission is measured.

In CNT toxicity studies, the focus has been on the perspective that the main pathways of CNTs into the body are through the respiratory system, skin, or gastrointestinal tract. In *in vitro* toxicity studies, the concentrations of tested CNTs also vary widely, from 1 to 400 μg/mL.^6^ These concentrations are representative of leachable levels. However, significantly higher concentrations may become relevant for surfaces fabricated with CNTs.

In 2006, Wörle-Knirsch et al. published research demonstrating that SWCNTs interact with MTT formazan crystals, leading to false results.^18^ They concluded that XTT, INT, and WST-1 tetrazolium salts did not interact with SWCNTs in the same way as MTT.^18^ However, it has later appeared that many toxicity assays do not work properly with CNTs.^19,20^ Consequently, we will investigate this issue more closely with SWCNTs and MWCNTs and six common cell viability assays: MTT (3-(4,5dimethylthiazol-2-yl)-2,5-diphenyl-2H-tetrazolium bromide), MTS (3-(4,5-dimethylthiazol-2-yl)-5-(3carboxymethoxyphenyl)-2-(4-sulfophenyl)-2H-tetrazolium), INT (2-(4-iodophenyl)-3-(4-nitrophenyl)-5-phenyl-2H-tetrazolium chloride), XTT (sodium 30-[1-(phenylaminocarbonyl)-3,4-tetrazolium]-bis (4-methoxy6-nitro) benzenesulfonic acid hydrate), WST-1 (4-[3-(4-iodophenyl)-2-(4-nitro-phenyl)-2H-5tetrazolio]-1,3-benzenesulfonate), and WST-8 (2-(2-methoxy-4-nitrophenyl)-3-(4-nitrophenyl)-5-(2,4-disulfophenyl)-2H-tetrazolium). The aim of our study is to determine whether other tetrazolium salt assays, in addition to the MTT assay, interfere with SWCNTs and MWCNTs. Another goal is to investigate the literature to evaluate how widely cell viability assays are used to study the toxicity of CNTs.

## 2 Materials and Methods

### 2.1 Interaction Studies

#### 2.1.1 Materials

SWCNTs (01RW03) were purchased from OCSiAl (Serbia). The diameter of Tuball SWCNTs is ≤2 nm, length >5 μm, and purity ≥94— ≤ 100%. Reported residuals for SWCNT include Cr < 350 ppm/Fe < 8000 ppm/Cu < 20 ppm/Ni < 350 ppm/Zn < 20 ppm. MWCNTs (PD30L5–20) were purchased from NanoLab (Massachusetts, USA). The diameter of MWCNTs is 30 ± 15 nm, length 5—20 μm, and purity >95%. Reported residuals for MWCNTs may include iron and sulfur. MTT formazan and INT formazan were purchased from Sigma-Aldrich. MTS Assay Kit was purchased from Abcam, XTT Cell Proliferation Assay Kit from Cayman Chemical, WST-1 Cell Proliferation Assay Kit from Cayman Chemical, and Cell Counting Kit −8 (WST-8) from Sigma-Aldrich.

#### 2.1.2 Formazan Reduction of the Dyes

Before conducting measurements with SWCNTs and MWCNTs, the MTS, XTT, WST-1, and WST-8 reagents in assay kits were reduced to a formazan product with yeast cells. The yeast mixture (200 mg/mL) was prepared in distilled water and then incubated at 37 °C for 4 h. Samples were prepared by adding the assay kit reagent (MTS, XTT, WST-1, or WST-8) in a 1:10 ratio, 0.1 M MES (Sigma-Aldrich) in a 1:10 ratio, yeast mixture in a 1:25 ratio, and the remaining volume filled with distilled water. After preparation, the samples were incubated at 37 °C overnight for the reduction process. The next day, the MTS, XTT, WST-1, and WST-8 formazan solutions were centrifuged at 2000 g for 5 min to extract samples from yeast cells. Supernatants were then used for measurements with SWCNTs and MWCNTs. A 0.5 mg/mL MTT and INT formazans were dissolved in DMSO (Sigma-Aldrich).

#### 2.1.3 Colorimetric Assays

0.5 mg/mL, 5 and 50 mg/mL SWCNTs and MWCNTs were weighed and then mixed into each formazan solutions in Eppendorf tubes. Formazan dye solutions without SWCNTs or MWCNTs were used as controls and were treated in the same way as the samples. Before the absorbance measurements, the SWCNT and MWCNT samples were centrifuged at 2000 g for 1 min, and 50 μL of supernatant was transferred to a 96-well plate with the controls. The supernatants were sufficiently clarified and free of CNT residues that could interfere with the absorbance readings. Absorbances were measured using Tecan Infinite F 200 Pro plate reader at 570 nm (MTT), 492 nm (MTS and INT) or 450 nm (XTT, WST-1 and WST-8). Measurements were taken at time points 0, 3, 6, 24, 48, and 72 h. Between measurements, samples and controls were incubated at 37 °C.

#### 2.1.4 Statistical Analyses

Each experiment was independently repeated three times, and all treatments were performed in triplicate per experiment. The average was calculated from controls and identically treated samples. The results were then normalized by comparing the sample values with the control values, assigning the controls a value of 1. Error bars represent the standard error of the mean (SEM). Molecular structures were created using ChemDraw Professional 22.2.0. Line graphs were plotted using Origin 2016.

### 2.2 Search Strategy of Literature Survey

The aim of the literature survey was to find out how widely different cell viability assays have been utilized in CNT toxicity studies since the study by Wörle-Knirsch et al. that highlighted the limitations of the MTT assay was published in 2006.^18^ The search was carried out in four-year periods from 2008 to 2023 using the Scopus database. Search terms consisted of combinations of key terms using the Boolean operators, ‘AND’ and ‘OR’. The search was limited to peer-reviewed articles, and the language was limited to English. Articles were independently searched by two authors (J. S. and S. V.).

Regarding the Scopus database, we employed the following request to identify relevant publications: ((“carbon nanotube” OR “carbon nanofiber” OR cnt OR swcnt OR dwcnt OR mwcnt OR graphene) AND (toxicity OR toxicology OR biocompatibility OR toxic OR cytotoxicity OR genotoxicity OR genotoxicology OR nanotoxicology OR nanotoxicity) AND (assay OR test)).

In our literature survey, we encompassed all six cell viability assay dyes (MTT, MTS, INT, XTT, WST-1, and WST-8) used in the experimental part. Furthermore, we included the Trypan Blue (TB) and Alamar Blue (AB) tests, which are also widely used methods for the evaluation of CNT toxicity.^6^ The following keywords were added to the above request in separate searches:

AND (mtt)

AND (mts)

AND (ldh OR int)

AND (xtt)

AND (wst-1)

AND (wst-8)

AND (“alamar blue” OR alamarblue)

AND (“trypan blue” OR trypanblue)

## 3 Results

### 3.1 Interference of Cell Viability Assay Dyes with SWCNTs and MWCNTs

**Figure 1:**
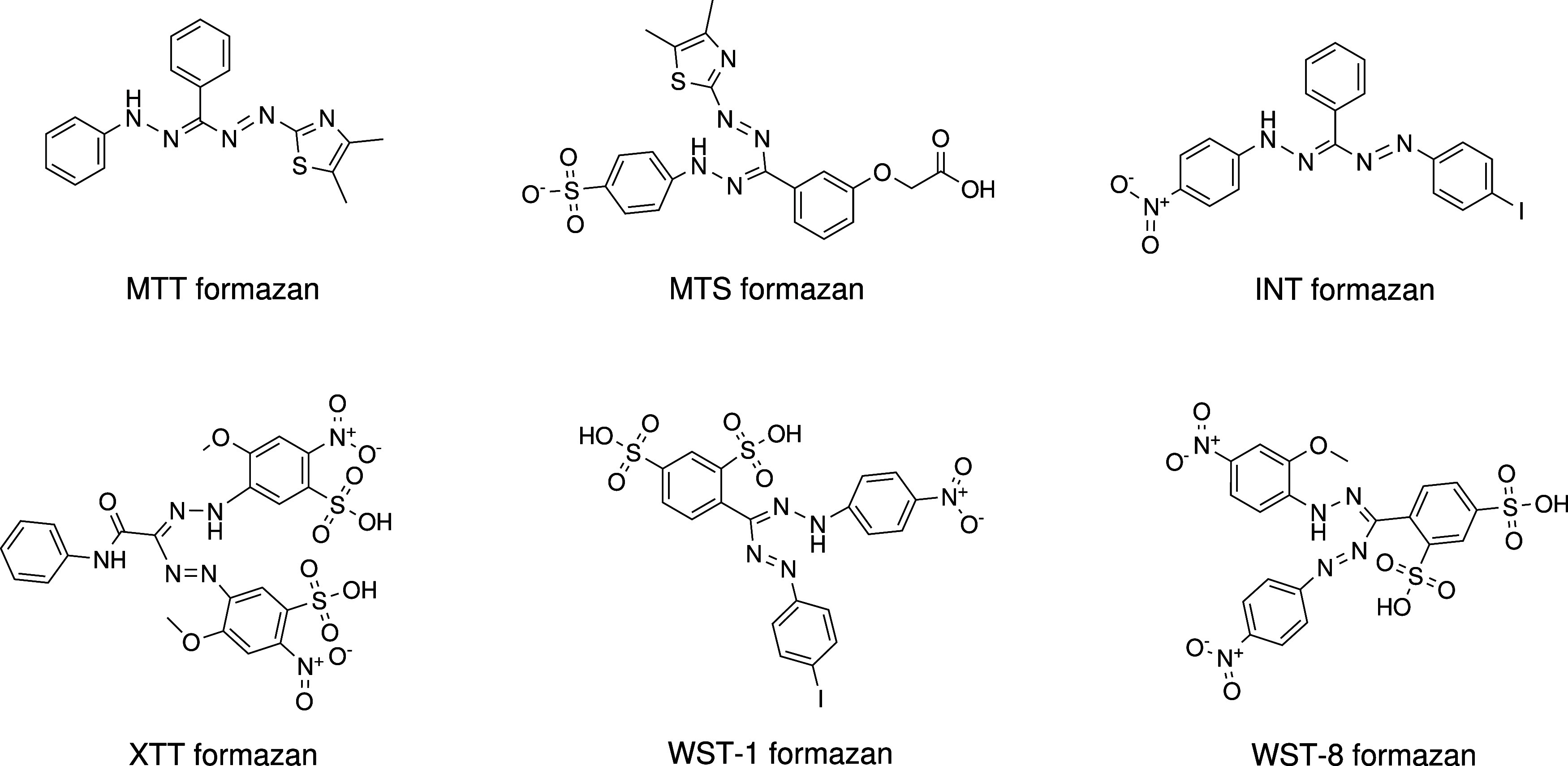
Chemical structures of MTT (1-(4,5-dimethylthiazol-2-yl)-3,5-diphenylformazan), MTS (4-(2-(3-(carboxymethoxy)phenyl)(-(4,5-dimethylthiazol-2-yl)diazenyl)methylene)hydrazineyl)benzenesulfonate), INT (1-(4-iodophenyl)-5-(4-nitrophenyl)-3-phenylformazan), XTT (4-methoxy-5-(2-(−1-(-(2-methoxy-4-nitro-5-sulfophenyl)diazenyl)-2-oxo-2-(phenylamino)ethylidene)hydrazineyl)-2-nitrobenzenesulfonic acid), WST-1 (4-(((4-iodophenyl)diazenyl)(2-(4-nitrophenyl)hydrazineylidene)methyl)benzene-1,3-disulfonic acid), and WST-8 (4-(-(2-(2-methoxy-4-nitrophenyl)hydrazineylidene)(-(4-nitrophenyl)diazenyl)methyl)benzene-1,3-disulfonic acid) formazans used in spectrophotometric measurements to investigate their interference with SWCNTs and MWCNTs.

To investigate interactions between CNTs and cell viability assay dyes, we incubated SWCNTs and MWCNTs with six different formazan solutions in cell-free systems, as described in Section 2. In addition to the dye from the commonly used MTT assay, we selected dyes from five other cell viability assays, which are also tetrazolium salts and structurally similar (Figure 1). We chose three different concentrations (0.5 mg/mL, 5 mg/mL, and 50 mg/mL) to determine at which concentrations interactions occur. The adsorption of cell viability dyes onto MWCNTs and SWCNTs was observed spectrophotometrically. We evaluated the response at 0 h to confirm the immediate effect. The 3-h time point was selected based on the recommended incubation period specified in ISO 10993–5:2009, *Biological Evaluation of Medical Devices—Part 5: Tests for In Vitro Cytotoxicity*. Extended incubation periods were included to determine the saturation point of dye absorption.

MTT and INT formazans were commercially purchased, while the other formazans were generated through the reduction activity of yeast cells. Therefore, it was essential to confirm the successful extraction of formazan from the yeast cells by monitoring the stability of the control samples. (Figure S1).

#### 3.1.1 Comparison of SWCNTs and MWCNTs

**Figure 2:**
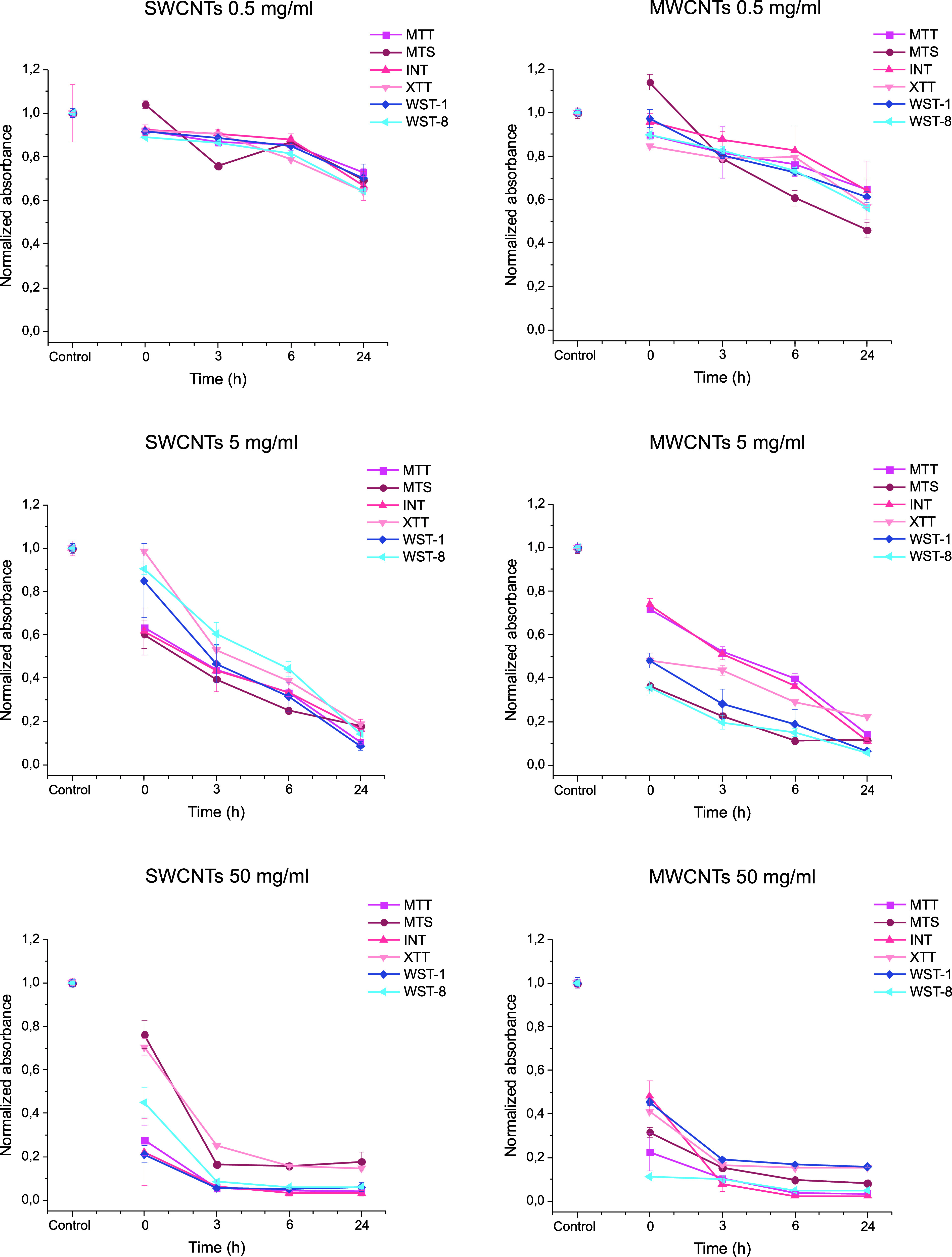
Interference of SWCNTs and MWCNTs at different concentrations (0.5, 5, and 50 mg/mL) affects the accuracy of six common cell viability assays (MTT, MTS, INT, XTT, WST-1, and WST-8 formazan). The absorbance of each formazan dye was measured at 0, 3, 6, and 24 h time points in cell-free systems. Absorbance values obtained from controls without CNTs, maintained under identical conditions to samples, were normalized to 1. Error bars represent the standard error of the mean.

The lowest tested concentration of SWCNTS and MWCNTS was 0.5 mg/mL, which immediately decreased the absorbance of the formazan upon addition (Figure 2). SWCNTs caused a decrease in absorbance on average to 93%, while MWCNTs caused a decrease of on average to 95%, compared to the control. The overall trend of the absorbance of each formazan dye was decreasing over time. After 3 h incubation, the usual measurement point in most assay protocols, the absorbances had decreased on average to 87% with SWCNTs, and to 82% with MWCNTs, compared to control. At 24 h, when the absorbance values had decreased on average to 68% and 58% compared to control, with SWCNTs and MWCNTs, respectively.

With higher concentrations, the trend was similar, but more pronounced. Addition of 5 mg/mL of SWCNTs or MWCNTs resulted in immediate decrease in the absorbance on average to 77% and 52% compared to control, with SWCNTs and MWCNTs, respectively. At 3h, the absorbances were decreased already on average to 48% and 36% with SWCNTs and MWCNTs, respectively. At 24 h the absorbance was on average only 14% and 12%, for SWCNTs and MWCNTs, respectively.

With the highest tested concentration, 50 mg/mL, the absorbance decreased immediately on average to 44% and 33% compared to control, with SWCNTs and MWCNTs, respectively. Most of the absorbance decrease had already occurred at 3 h, when the absorbances were decreased on average to 11% and 13% compared to control, with SWCNTs and MWCNTs, respectively.

Figure S2 shows average of all dyes in the tested CNT concentrations. At the lowest concentration of 0.5 mg/mL, absorption continued to decrease after 24 h. In contrast, at 5 mg/mL, CNT adsorption reached saturation at 24 h, indicated by the dye absorbance remaining stable after 24 h. For the highest concentration of 50 mg/mL, this stabilization occurred as early as 3 h. On average, the MWCNTs decreased the absorbance of the formazan dyes slightly more than the SWCNTs.

#### 3.1.2 Comparison of Different Dyes

**Figure 3:**
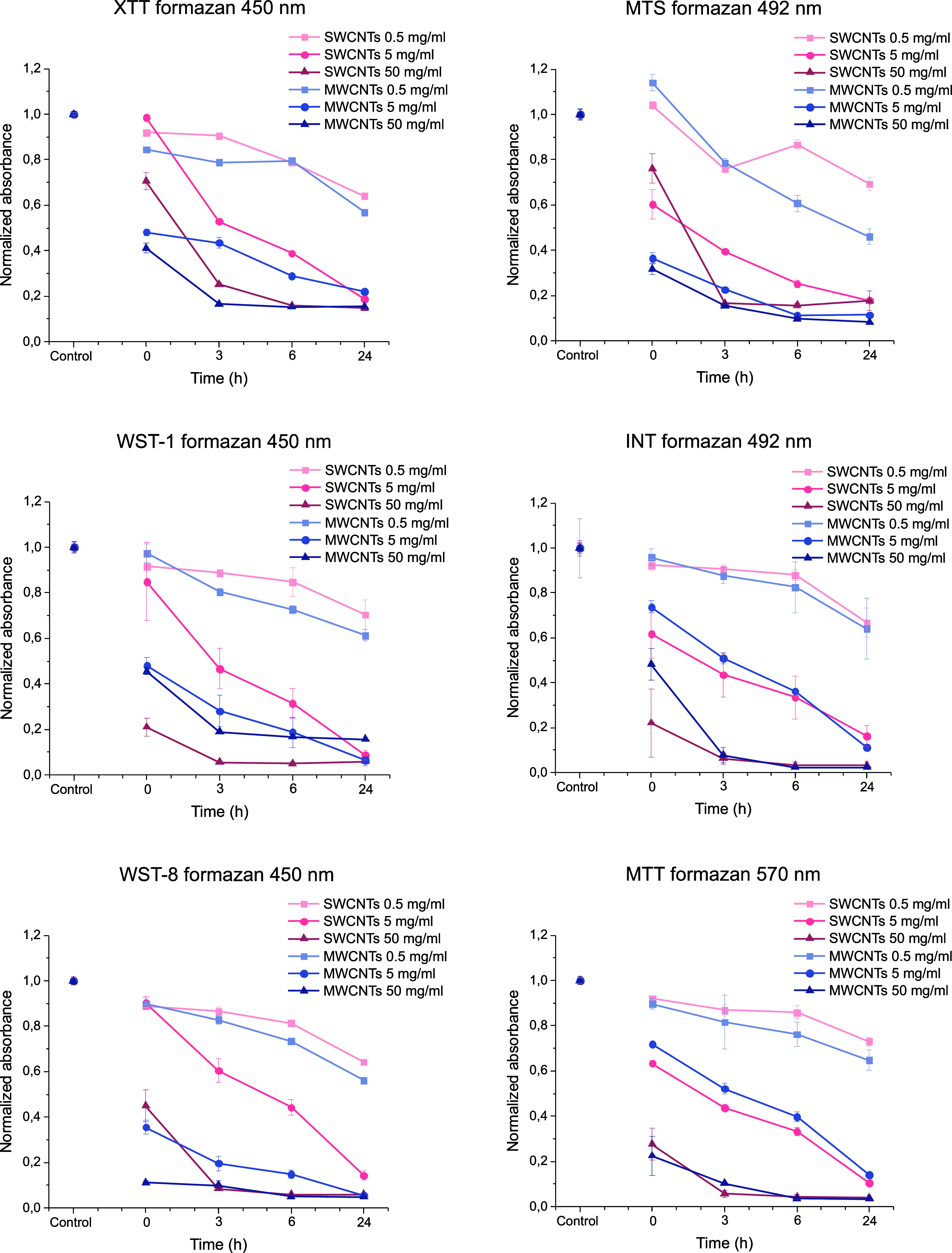
Comparison of how SWCNTs and MWCNTs at different concentrations (0.5, 5, and 50 mg/mL) interfere with the accuracy of six common cell viability assays (XTT, WST-1, WST-8, MTT, MTS, and INT) in cell-free systems. The absorbance of each formazan was measured at 0, 3, 6, and 24 h time points at wavelengths of 450 nm (XTT, WST-1, WST-8), 570 nm (MTT), or 492 nm (MTS, INT). Absorbance values obtained from controls without CNTs, maintained under identical conditions to samples, were normalized to 1. Error bars represent the standard error of the mean.

Figure 3 presents the same data as Figure 2, but reorganized to facilitate the comparison of different dyes. On the left-side panel, the absorbance for the XTT, WST-1, and WST-8 assays is measured at 450 nm. On the right-side panel, the absorbance for the MTT assay is measured at 570 nm, while the MTS and INT assays are measured at 492 nm. The other formazans, apart from MTT and INT, were produced by yeast cell reduction. MTT and INT formazans were commercially purchased, while the other formazans were generated through the reduction activity of yeast cells.

MTT and INT formazans perhaps showed the least difference between SWCNTs and MWCNTs (Figure 3), and clearest concentration dependence in the absorbance reduction. Aside from this, no significant differences were observed between the various dyes or dye clusters measured at specific wavelengths. In all tested formazan dyes and with both types of CNTs, SWCNTs and MWCNTs, a noticeable decrease in absorbance was observed at different concentrations compared to the controls. The higher the concentration of SWCNTs or MWCNTs in the formazan solution, the faster and more significantly adsorption occurs over time. That indicates interaction between the dye molecules and CNTs while absorbances in the control dyes without CNTs did not decrease compared to the beginning of the experiment (Figure S1).

### 3.2 Literature Survey of *In Vitro* Toxicity Studies

**Figure 4:**
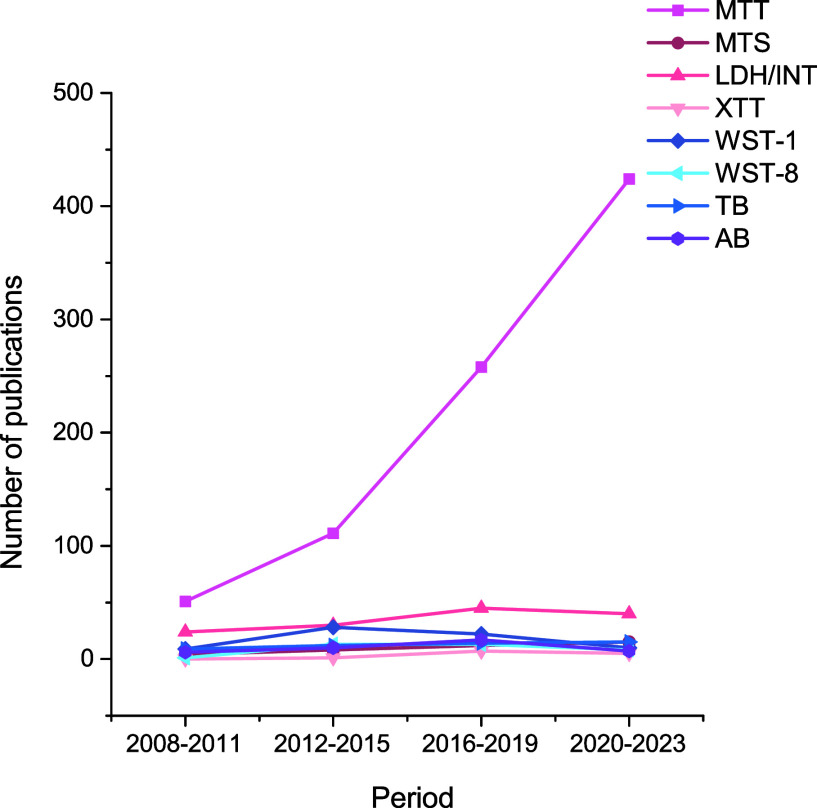
Number of published *in vitro* carbon nanomaterial toxicity studies including cell viability assays MTT, MTS, INT, XTT, WST-1, WST-8, Trypan Blue (TB), and Alamar Blue (AB), based on Scopus database search. The search was carried out in four-year periods, starting from 2008 until 2023.

Studying the toxicity and health risks of CNTs became topical when they began to be used extensively in industry since the 1990s. Figure 4 shows that from the year 2008 to 2023, the use of MTT assay in carbon nanomaterial toxicity studies has increased explosively compared to other cell viability assays, although it was the MTT assay that was first found to cause interference with CNTs.^18^ The use of the MTT assay has increased more than 8-fold during the studied time period, while the use of other assays (MTS, INT, XTT, WST-1, WST-8, TB, and AB) has remained fairly low and consistent.

## 4 Discussion

Although it was observed in several studies nearly two decades ago that the MTT assay is an unreliable method for studying the toxicity of CNTs, our results indicate that it remains the most commonly used cell viability assay for this purpose (Figure 3).^18,20,21^ Interestingly, the use of MTT has even increased significantly compared to other dyes. Chetyrkina et al. also showed that commonly used methods for evaluating the toxicity of CNTs in dispersion *in vitro*, in descending order, are MTT, LDH, Trypan Blue, WST-1, Alamar Blue, and MTS-8.^6^

According to the study by Wörle-Knirsch et al., XTT, LDH, and WST-1 assays appeared to perform better than the MTT assay.^18^ MTT, INT in the LDH assay, XTT, and WST-1 are all tetrazolium salts, but the difference is that in the MTT assay the end product, MTT formazan, is not water-soluble, unlike the end products of other assays. Wörle-Knirsh et al. observed that SWCNTs bind MTT–formazan crystals and stabilize their chemical structure when the crystals cannot be solubilized in solvents, which could be crucial for adsorption.^18^ However, it was later been found that other toxicity assays, in addition to MTT, can also interfere, suggesting there must be other reasons for interactions than the solubility.^19,20^

In the cytotoxicity assays, the cell viability is calculated as a percentage of control:
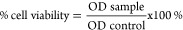


A sample is considered cytotoxic if the viability value is <70%*.* As our results show, all tested tetrazolium salt assays—MTT, MTS, INT, XTT, WST-1, and WST-8—interacted with both SWCNTs and MWCNTs and were adsorbed onto them, resulting in decreased absorbance (Figure 2). At a concentration of 5 mg/mL and a typical testing time of 3 h, this interference would lead the tests to falsely indicate a cytotoxic effect, as the viability values were clearly below 70%. The interference was significant also with the smaller tested concentration of 0.5 mg/mL, where the absorbance values had decreased to 87% with SWNTS, and to 82% with MWCNTs, compared to control at 3 h. Over an extended testing period of 24 h, even the lowest concentration of 0.5 mg/mL would falsely indicate a cytotoxic effect.

Overall, the interfering effect of MWCNTs was stronger than that of SWCNTs. There can be several reasons for this: MWCNTs have a larger surface area, a higher number of adsorption sites, more defects and functional groups, a lower tendency to aggregate, and the ability to utilize both inner and outer surfaces for adsorption. Moreover, CNTs, especially when in suspension, can absorb light at various wavelengths, which may overlap with the absorbance of the formazan product generated in the assays.^22^MWCNTs generally have a higher overall light absorption in the visible range because they absorb light continuously across a broad spectrum, albeit less selectively. This overlap could lead to inaccurate readings of cell viability, either by artificially increasing or decreasing the apparent absorbance values. Chirality sorted SWCNTS may have specific interference with absorbance-based assays, as they can have very efficient and specific light absorption at certain wavelengths.

There were no significant differences between the different dyes. MTT and INT formazans exhibited minimal differences in behavior between SWCNTs and MWCNTs and displayed the most pronounced concentration-dependent reduction in absorbance. Since these formazans were purchased rather than produced by yeast cell reduction, there were fewer sources of experimental error.

It is still partly unclear what exactly the interactions between CNTs and assay dye molecules are, but there are a few suggestions related to physicochemical characteristics of CNTs and nanoparticles overall. These include a large surface area, which leads to increased adsorption capacity, optical properties that can interfere with light absorption detection systems, increased catalytic activity, and magnetic properties.^23−25^ The main conclusion regarding interference with cell viability dyes is the strong adsorption capacity of CNTs. CNT -based adsorbents are gaining considerable interest in both research and industrial sectors because of their expansive surface area, cylindrical hollow structure, and abundant mesopores.^26^ It has been estimated that types of CNT products and surfactants used to suspend CNTs can affect interactions due to treatment procedures that modify the surface chemistry.^21^ The type, size, shape, aggregation and impurities of the CNTs can also influence their toxicity.^7,32−34^ However, it is likely that these same factors affect the surface chemistry and adsorption of the viability agent. To fully understand the nature of dye-CNT interactions, a detailed characterization of the CNTs is necessary.

The adsorption mechanisms primarily entail van der Waals forces, π–π stacking, hydrophobic interactions, hydrogen bonding, and electrostatic interactions.^26^ In carbon nanostructures, especially π–π interactions, are the dominating supramolecular forces, and CNTs interact with biomolecules through π-stacking of sp^2^ bonds.^27^ There is evidence that many biological molecules have a strong affinity for nanoparticles.^28^ Molecular geometry and charge have been found to be pivotal factors influencing the interactions between organic dyes and MWCNTs.^29^ Molecules with planar structures and high charge load seems to be favored for the adsorption. This type of interaction can likely occur between CNTs and molecules in cell viability assays. Figure 1 shows that all tested dyes have multiple benzene groups, making them planar and charged molecules.

Returning to the characteristics of the cell viability assay molecules, CNTs have unique electronic properties that might interact with the reagent itself, not just with the formazan forms. They could potentially reduce dyes directly, leading to altered levels of formazan formation, which would affect the assay’s accuracy.^21^

In addition to tetrazolium salts, CNTs have been found to cause interference with other common cell viability assays. The Alamar Blue assay, also a reduction assay based on resazurin salt, has been found to interact with CNTs.^19,35,36^ Another common dye, Neutral Red, which separates viable and dead cells by being absorbed into viable cells, has also been found to interact with CNTs.^19,20^ Davoren et al. implemented *in vitro* toxicity evaluation of SWCNTs on A549 cells using the MTT, Alamar Blue, and Neutral Red (NR) assays, and some interference was observed in these assays.^20^ Later, Casey et al. found interactions of varying degrees between SWCNTs and all MTT, WST-1, Coomassie Blue, Alamar Blue, and Neutral Red assay dyes in spectroscopic analysis.^19^

The challenges related to cell viability assays in the toxicity studies of CNTs are a serious problem and do not seem limited to only CNTs and absorbance-based assays.^23,37,38^ Other carbon-based nanomaterials and nanoparticles have also shown invalid results with cell viability assays MTT,^24,36,39−42^ Neutral Red,^36^ LDH^24,39,43^ and WST-8.^41^ Since absorbance-based and fluorescence-based assays were shown to interfere with CNTs, Szymański et al. carried out luminescence-based tests with MWCNTs.^17^ However, this study also showed that luminescence-based tests produce false results when evaluating the cytotoxicity of CNTs. Therefore, it is essential to carefully examine which *in vitro* methods are truly suitable for toxicity studies of CNTs. In toxicity studies, the interaction between nanoparticles and assay components should be controlled also without cells in addition to positive and negative controls observing possible interference. Although colorimetric and fluorescence-based measurements are common in toxicity studies, interference is rarely controlled in any way.^30,37,44^

Previous findings suggesting that water-soluble assays perform better may stem from the fact that assays relying on insoluble formazan, such as MTT and INT assays, are more challenging to optimize. The insoluble formazan crystals produced by the assay can be difficult to dissolve uniformly, requiring additional steps such as the use of solvents (e.g., DMSO or isopropanol). This can lead to variability in results and affect assay sensitivity, especially when working with test materials that interact with the solvent.

Despite maintaining consistent yeast cell numbers and incubation times, variations in the conversion of the dye to formazan may occur. This also complicates the comparison between different dyes, as the concentrations of commercially available dyes (such as MTT formazan and INT) and biologically produced formazan forms (MTS, XTT, WST-1, WST-8) are not equivalent. In biological processes, the final concentration of formazan can vary, further adding to the challenge. Overall, the adsorption effect becomes particularly significant when the dye concentration is relatively low, and the CNT concentration is relatively high, as adsorption has a more pronounced impact under these conditions.

While the challenges observed here are significant, they can be manageable. The assay system, including the detection method, has to be thoroughly evaluated for assay-test material interactions. It is essential to conduct appropriate control/validation experiments to identify potential nonspecific interactions that could impact the interpretation of the assay results. If interactions are present, researchers must evaluate their impact on the assay to determine whether they invalidate the biological observations or if analytical methods can accurately quantify the effects.

Moreover, while considering the assay reliability, the assay-test material interaction is not the only confounding variable. Other aspect to consider include the number of cells seeded, assay concentration, incubation time, serum starvation conditions, composition of the cell culture media, release of intracellular contents, and the extrusion of formazan into the extracellular space.^31^ These are often overlooked variables, and the lack of their optimization causes variability in the protocols and, consequently, in the results. In our cell-free system, we focused only on the assay-test material interaction. In conclusion, it is clear that to accurately assess the toxicity of nanomaterials, at least two independent, validated *in vitro* assays should be employed.

## 5 Conclusions

Interference of the cell viability assay components with CNTs is a serious issue, and alternative methods for measuring the impact of CNTs on cell viability should be considered, especially with high CNT concentrations. The growing number of CNTs are used in biological environments, and most of the research uses cell viability assays or their derivates in *in vitro* toxicity studies. Despite the issue of MTT being published nearly 20 years ago, researchers are not aware of the issue as evidenced by the high number of articles still published. The previous study concluded that XTT, INT, and WST-1 tetrazolium salts did not interact with SWCNTs in the same way as MTT. Our study revealed that the interference of all six tested formazan dyes with both SWCNTS and MWCNTs, poses a significant challenge with high CNT concentrations, making them less suitable for accurately assessing the toxicity of CNTs at high concentrations, such as surfaces fabricated with CNTs. The interference effect of MWCNTs was slightly stronger than SWCNTs, but there were not significant differences between dyes. Overall, all the dyes caused a strong decrease of absorbance with both types of CNTs indicating adsorption. In toxicity studies, a decrease of absorbance indicates toxicity, which can lead to false results with CNTs. Due to the interference effect, CNT samples of 5 mg/mL would be interpreted cytotoxic within the typical testing time of 3 h. Knowledge and understanding of interactions between CNTs and molecules, and careful consideration of control/validation experiments, are needed to find more reliable methods to evaluate the toxicity of CNTS in biological environments. When conducting cytotoxicity assays with carbon-based nanomaterials, we recommend testing specific incubation times and concentrations with formazan to identify potential dye-CNT interactions.

## Supporting Information Available

Absorbance of controls; average absorbance of all the tested dyes (PDF)
