# Supplementary material for: Save Your Tears for the Toxicity Assays—Carbon Nanotubes Still Fooling Scientists
Source: ACS Omega. 2025 Feb 3;10(6):5554–62. doi: 10.1021/acsomega.4c08211 (PMC11840583; doi:10.1021/acsomega.4c08211)
Supplement: Supplementary file 1 — ao4c08211_si_001.pdf [file ao4c08211_si_001.pdf]

# Supplementary material

## Save Your Tears for the Toxicity Assays — Carbon Nanotubes Still Fooling Scientists

Johanna Suni[1], Salli Valkama[1], and Emilia Peltola[1*]

[1]Department of Mechanical and Materials Engineering, University of Turku, Turku 20500, Finland

[*] Corresponding author: emilia.peltola@utu.fi

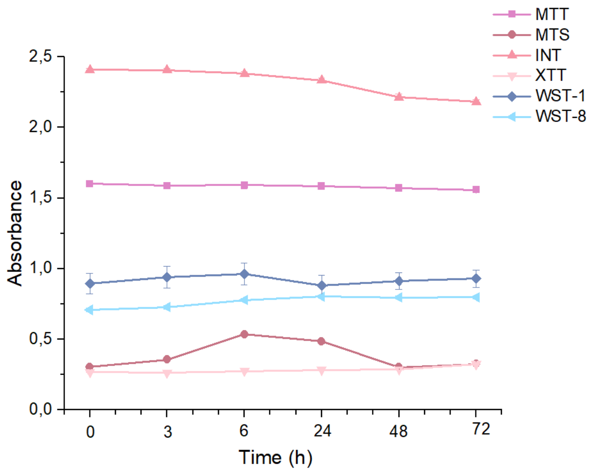


**Figure S1**. Absorbance of control of each cell viability assay dye measured at 0 h, 3 h, 6 h, 24 h, 48 h, and 73 h time points. Controls are dyes without CNTs, maintained under identical conditions to samples. Error bars represent the standard error of the mean.

MTT and INT formazans were purchased, while the others were generated through yeast cell reduction. Successful formazan extraction was confirmed by monitoring the stability of control samples. Although the other dyes remained highly stable, MTS showed a slight increase in absorbance at 6 and 24 hours (Fig. S1), suggesting incomplete extraction of yeast, with some cells continuing to metabolize MTS into MTS formazan. Despite this, the overall absorbance trend of MTS formazan decreased in the presence of CNTs, similar to the other dyes.

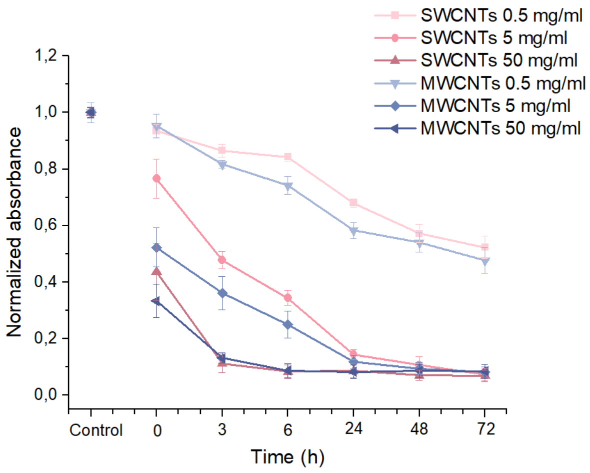


**Figure S2**. Average interference of all cell viability assay dyes in different concentration of SWCNTs and MWCNTs. Absorbance was measured at 0 h, 3 h, 6 h, 24 h, 48 h, and 73 h time points. Absorbance values obtained from controls without CNTs, maintained under identical conditions to samples, were normalized to 1. Error bars represent the standard error of the mean.
